# Supplementary material for: Nance-Horan-syndrome-like 1b controls mesodermal cell migration by regulating protrusion and actin dynamics during zebrafish gastrulation
Source: Commun Biol. 2025 Feb 28;8:328. doi: 10.1038/s42003-025-07689-6 (PMC11871229; doi:10.1038/s42003-025-07689-6)
Supplement: Supplementary file 1 — Supplementary Information [file 42003_2025_7689_MOESM1_ESM.pdf]

Supplementary Materials and Figures

Nance-Horan-Syndrome-like 1b controls mesodermal cell migration by regulating protrusion and actin dynamics during zebrafish gastrulation.

Sophie Escot, Yara Hassanein, Amélie Elouin, Jorge Torres-Paz, Lucille Mellottee, Amandine Ignace, Nicolas B. David

Figure S1: **Epiboly and mesoderm front progression in *nhsl1b* knockdown.**

Figure S2: *nhsl1b* expression levels need to be fine-tuned for proper mesodermal migration.

Figure S3: Nhsl1b knockdown using CRIPSR/Cas13d.

Movie 1: Lateral mesoderm migration in MO Control and MO Nhsl1b. Time-lapse imaging of *Tg(tbx16:EGFP)* embryos injected with MO Control or MO Nhsl1b, *LacZ* (control) or *nhsl1b* mRNAs. *nhsl1b* knockdown and overexpression slows lateral mesodermal migration Time interval 2 min; scale bar 50 µm.

Movie 2: Tracking of mesodermal cells. Tracking of mesodermal cell nuclei, labelled with H2B-mCherry, in *Tg(tbx16:EGFP)* embryos injected with Control or Nhsl1b Morpholinos. White spots show the nuclei and tracks are represented in yellow.

Movie 3: Time lapse imaging of Nhsl1b-mNeongreen localisation at the tip of actin-rich protrusions. Live imaging of Nhsl1b-mNeongreen and Lifeact-mCherry expressing mesodermal cells, plated on a coverslip. Time interval 1,3 sec; scale bar 5 µm.

Movie 4: High temporal resolution imaging of actin flows in MO Control and MO Nhsl1b mesodermal cells. Time-lapse showing growing protrusions in MO Control and MO Nhsl1b injected mesodermal cells expressing Lifeact-mNeongreen. Time interval 1,3 sec; scale bar 5 µm.

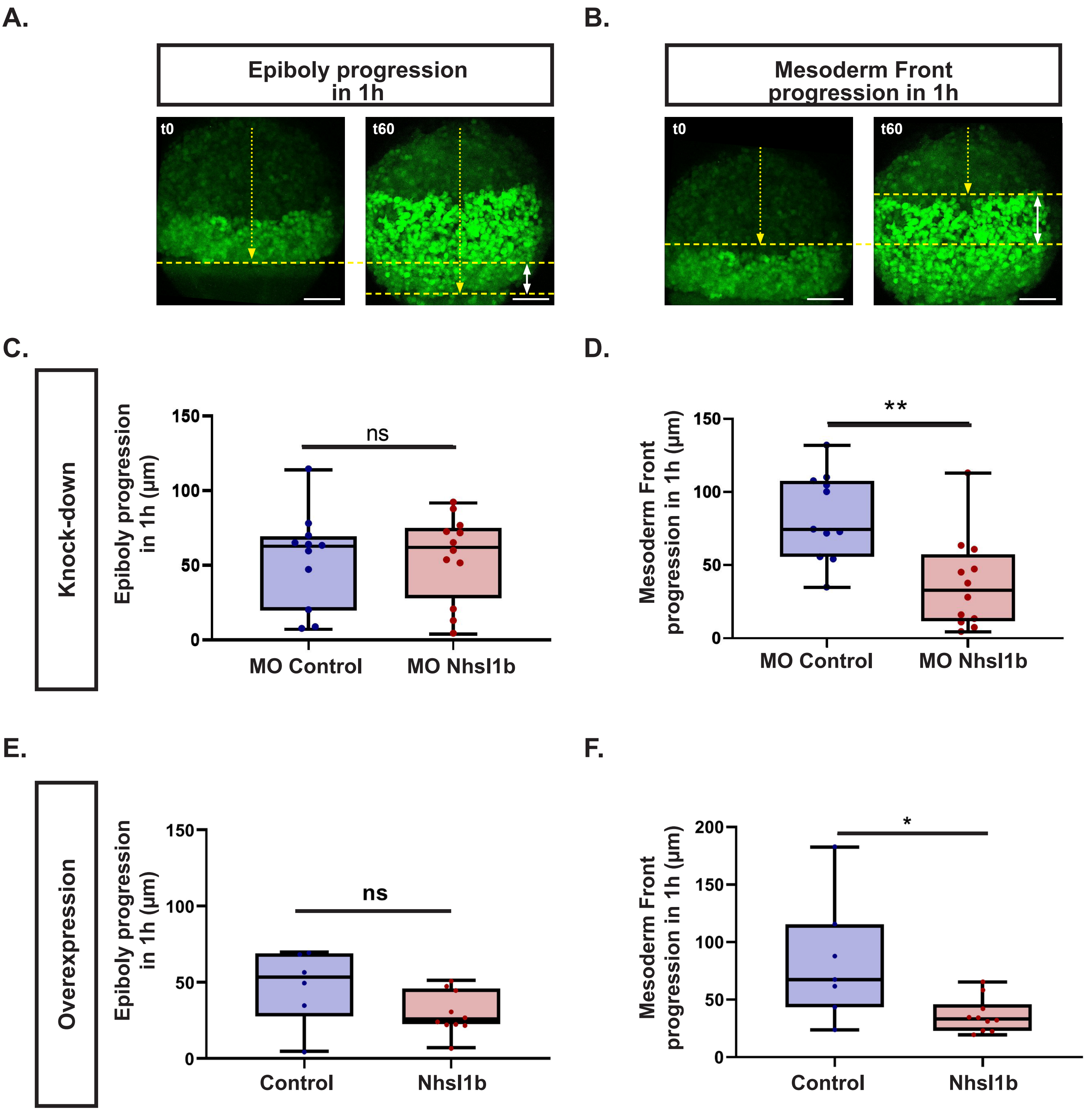

Figure S1: Epiboly and mesoderm front progression in nhsl1b knockdown.
(A) Epiboly progression between early gastrulation (60% epiboly stage) and one hour later. The distance between the animal pole and the embryonic margin was measured at the two time points (yellow dotted arrow), and epiboly progression was calculated as the difference between these measurements (white double arrow). (B) Progression of the mesoderm front between early gastrulation (60% epiboly stage) and one hour later. The distance between the animal pole and the mesoderm front was measured at the two time points (yellow dotted arrow), and progression of the mesoderm front was calculated as the difference between these measurements (white double arrow). (C) Quantification of the epiboly progression. Mann-Whitney test. p-value: MO Control vs MO Nhsl1b: 0.6947 ns. (D) Quantification of the mesoderm front progression. Mann-Whitney test. p-value: MO Control vs MO Nhsl1b: 0,0028 **. (C-D) MO Control (n=11) or MO Nhsl1b injected embryos (n=12). (E) Quantifications of the epiboly progression. Mann-Whitney test. p-value: Control vs Nhsl1b: 0,0934 ns. (F) Quantification of mesoderm front progression. Mann-Whitney test. p-value: Control vs Nhsl1b: 0,0312 *. (E-F) lacZ (control) (n=6) or nhsl1b (n=10) injected embryos. Scale bars 100 µm.

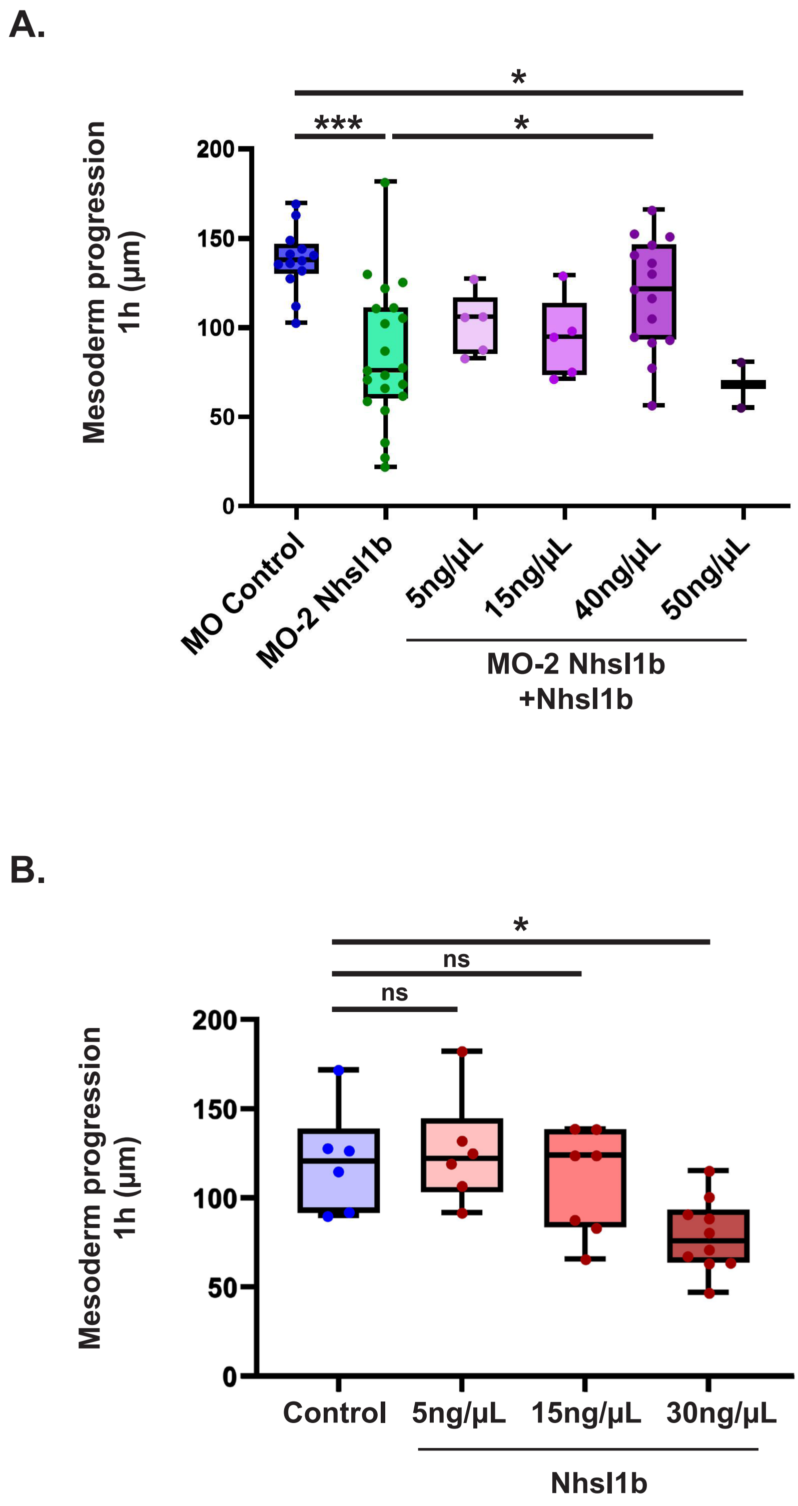

Figure S2: nhsl1b expression levels need to be fine-tuned for proper mesodermal migration.
(A) Rescue of nhsl1b knockdown (MO-2) phenotype on mesoderm progression by co-injecting different concentrations of nhsl1b mRNA. Only co-injection of 40ng.µL^{-1} of nhsl1b mRNAs restored mesoderm progression. Kruskal-Wallis test followed by Dunn's multiple comparison test, adjusted p-values: MO Control vs MO-2 Nhsl1b: <0,001 ***; MO Control vs MO-2 Nhsl1b + 50ng.µL^{-1} Nhsl1b: 0,0364 *; MO-2 Nhsl1b vs MO-2 nhsl1b + 40ng.µL^{-1} Nhsl1b: 0,0208 *. (B) Dose-response analysis of nhsl1b mRNA overexpression effects on mesoderm progression. Quantification of the lateral mesoderm progression in control embryos and embryos injected with different concentrations of nhsl1b mRNA. Low doses (5 and 15 ng.µL^{-1}) had no significant effect of mesoderm progression, 30 ng.µL^{-1} being the lowest dose inducing an effect. This is slightly lower than the 40ng.µL^{-1} used in the rescue experiments, as it here comes in addition to the endogenous expression of nhsl1b. Kruskal-Wallis test followed by Dunn's multiple comparison test, adjusted p-values: Control vs Nhsl1b 5ng.µL^{-1} >0.9999; Control vs Nhsl1b 15ng.µL^{-1} >0.9999; Control vs Nhsl1b 30ng.µL^{-1} 0.0269.

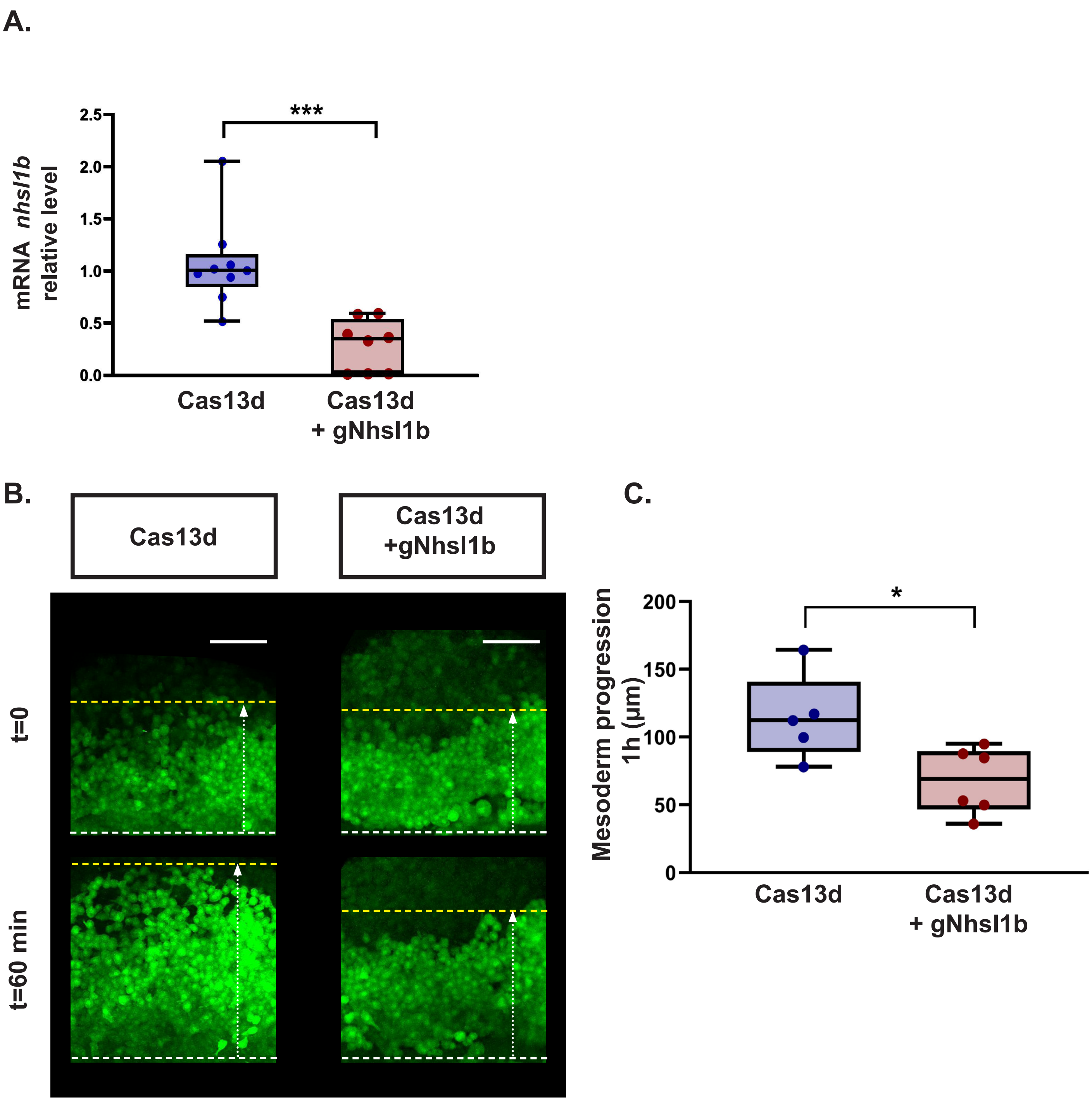

Figure S3: Nhsl1b knockdown using CRIPSR/Cas13d.
(A) RT-qPCR analysis showing levels of nhsl1b mRNAs in embryos during gastrulation (9 hours post fertilisation), injected with cas13d mRNA alone or cas13d mRNA and a mix of 3 gRNAs targeting nhsl1b. Results are shown as the averages ± standard error of the mean from three independent experiments. Cdk2ap mRNA was used as normalization control. Mann-Whitney test. p-value: Cas13d vs Cas13d + gNhsl1b: 0,0003***. (B, left) Representative lateral views of Tg(tbx16:EGFP) embryos, at early gastrulation (60% epiboly; t=0) and 1 hour later in embryos injected with cas13d mRNA alone or cas13d mRNA and nhsl1b gRNAs. Yellow dashed lines indicate the positions of the embryonic margin and of the front of the migrating mesoderm. Mesoderm progression was measured as the distance between these two lines. Scale bar 100 µm. (B, right) Quantification of the lateral mesoderm progression in cas13d injected embryos (n= 5), and cas13d + nhsl1b gRNAs injected embryos (n=6). Mann-Whitney test. p-value: Cas13d vs Cas13d + gNhsl1b: Cas13d vs Cas13d + gNhsl1b: 0,0303*.
